# Coeliac Disease in Children—A Clinical Review Including Novel Treatment Agents

**DOI:** 10.3390/medicina60101650

**Published:** 2024-10-09

**Authors:** Chloe Corlett, Astor Rodrigues, Madhur Ravikumara

**Affiliations:** 1 Department of Gastroenterology, Children’s Hospital Oxford, Oxford University Hospitals NHS Trust, Oxford OX3 9DU, UK; chloe.corlett@ouh.nhs.uk (C.C.); astor.rodrigues@ouh.nhs.uk (A.R.); 2 Department of Gastroenterology, Perth Children’s Hospital, Perth, WA 6009, Australia

**Keywords:** coeliac disease, children, gluten-free diet, novel therapies

## Abstract

Coeliac disease (CD) affects almost of 1% of the population, yet remains undiagnosed in the majority. Though the demonstration of enteropathy in duodenal biopsy was traditionally the essential criterion for the diagnosis of coeliac disease, the guidelines published by the European Society of Paediatric Gastroenterology and Nutrition (ESPGHAN) in 2012, and revised in 2020, paved the way to a no-biopsy approach to diagnosis. In a select group of children meeting certain criteria, a definitive diagnosis of CD can now be made without the need for duodenal biopsies. This is being increasingly applied in clinical practice. It is well established that untreated coeliac disease is associated with several chronic adverse health conditions. At present, a strict gluten-free diet remains the only effective treatment for CD. The advances in our understanding of the pathogenesis of CD have led to a search for alternative treatment agents. Several investigational agents are in various phases of clinical trials at present. In this review, we outline the clinical aspects of coeliac disease and summarise various investigational treatment agents.

## 1. Introduction

Coeliac disease (CD) is an autoimmune disease characterised by an immunological response to the ingestion of gluten in genetically susceptible individuals. Increased knowledge and awareness of the condition, in combination with improved diagnostic tests, has transformed our understanding of CD from a rare enteropathy to a common multisystem disorder [1].

Gluten is a prolamin, a seed storage protein found in several cereal grains such as wheat, barley, and rye. The key elements in the pathogenesis of CD are gluten intake, autoantibody formation against tissue transglutaminase (tTG-IgA), and the genotypes HLA-DQ2/DQ8. Other likely pathogenetic factors that play a part are the loss of intestinal barrier function, a gluten-induced proinflammatory innate immune response, an inappropriate adaptive immune response, and an unbalanced gut microbiome [2].

## 2. Epidemiology

The estimated prevalence of CD worldwide is approximately 1%—based on population-based serological surveys [3,4]. Diagnostic rates have increased due to a combination of increased awareness and improved accuracy of serological tests; despite this, up to 90% of cases remain undiagnosed [5]. The pooled prevalence in first-degree relatives was 7.5% [6], and the screening of family members is an important part of the management process.

The mode of delivery, protective effects of breast milk, and age of weaning/gluten introduction in infants have not been substantiated as factors that affect the risk of developing CD. There is ongoing research regarding whether gastrointestinal gut infections such as rotavirus increase the risk of coeliac disease [2].

## 3. Clinical Manifestations

Gastrointestinal symptoms and extra-intestinal symptoms are varied and are outlined in the Table 1.

Historically, younger children tend to present with classical symptoms of abdominal pain, diarrhoea, anorexia, abdominal distention and, if the diagnosis is delayed, behavioural changes and malnutrition. Older children may develop gastrointestinal symptoms, depending on the amount of gluten ingested; otherwise, their presentation can often be mild and non-specific [2].

CD should also be considered in asymptomatic children with associated conditions, as outlined in the Table 2 as the risk of CD is greater than that of general population in this group of children.

The presentation of CD has changed significantly in the last few decades. Paediatric patients diagnosed in recent times seemingly have less severe symptoms than 20 years ago [7]. Classical symptoms are rarely seen, and often, presentation is atypical, mild, or non-specific [8]. Almost 25% of children with CD are diagnosed by targeted screening and report no symptoms [9]. Presentation with extra-intestinal manifestations has been seen more commonly than with GI symptoms [10,11]; the cause of this is unclear, but it is potentially related to the increased awareness and greater accuracy of diagnostic testing. Short stature is one of the most common extra-intestinal manifestations in children, seen in 10–47.5% [12].

## 4. Diagnosis

The diagnosis of coeliac disease is based on the combination of symptoms, serological testing, and duodenal biopsy.

### 4.1. Serologic Screening for Possible CD

Testing for CD in symptomatic children and asymptomatic children with associated conditions is carried out via an initial screening test for serum tTG-IgA whilst the child is on a gluten-containing diet. False negatives can occur in the context of Selective IgA deficiency; therefore, total IgA should be tested at the same time. Selective IgA Deficiency can be formally defined on the basis of serum IgA < 0.07 g/L on two separate occasions in a child aged 4 years or older [13]. In such individuals, positive IgG-based serologic tests may support the presence of CD (tissue transglutaminase (tTG-IgG), endomysial antibody (EMA-IgG), or deamidated gliadin peptide (DGP-IgG)), but because these are less specific than IgA assays, it is suggested that a diagnosis can only be securely established on the basis of duodenal biopsy and histopathology.

The presence of gastrointestinal symptoms related to gluten intake without positive serology may be consistent with diagnoses of either Non-Coeliac Gluten Sensitivity or Seronegative CD. Formal differentiation of these requires duodenal biopsy, but a pragmatic approach may be indicated if symptoms are mild, and specialist input should be sought. Clinical suspicion of a malabsorptive enteropathy should prompt referral for the consideration of endoscopy regardless of CD serology, since this can be caused by a range of conditions.

### 4.2. Confirming the Diagnosis without Duodenal Biopsies

Although CD diagnosis has historically relied on the demonstration of enteropathy in the duodenal biopsy, the ESPGHAN guidelines published in 2012 paved the way, for the very first time, to diagnosing CD without the need for duodenal biopsy in a subset of children, with high diagnostic accuracy. In the initial guidelines published in 2012, it was stated that a diagnosis of CD could be made without duodenal biopsy in symptomatic children who had tTG-IgA titres more than 10 times the upper limit of normal and a positive anti-endomysial antibody-IgA (EMA-IgA) in a second blood sample, in the presence of positive HLA risk alleles (DQ2 and/or DQ8) [14].

Asymptomatic children with positive coeliac serology detected on the basis of screening (e.g., type 1 diabetes, a first-degree relative with the disease) were initially excluded from this pathway. In subsequent years, numerous prospective and retrospective studies evaluated the performance of this guidance, which led to further changes. The key diagnostic indicator of a highly elevated tTG-IgA more than 10 times the upper limit of normal demonstrated a similar assured positive predictive ability in asymptomatic children, as well as in those with symptoms. On the basis of this, ESPGHAN revised their guidelines in 2020 [15] to include asymptomatic children and retracted the need for HLA typing. Thus, according to the revised guidelines, a diagnosis of CD can be secured in a child who has tTG-IgA more than 10 times the upper limit of normal and a positive EMA-IgA in a second blood sample, following discussion with the family, who understand and agree with a non-biopsy diagnosis [15]. It is essential that clinicians diagnosing CD using the no-biopsy approach have a clear understanding of which assay their diagnostic laboratory is using and how multiples of upper limit of normal relate to duodenal architectural changes in their centre. The decision on whether or not to undertake endoscopic biopsy to establish a diagnosis of CD should be taken with parents/carers and the patient (if appropriate) as a shared decision-making process. While tTG-IgA ≥ 10 times the upper limit of normal appears to have very high positive predictive value in symptomatic patients, this may be reduced in those who are asymptomatic. Current evidence suggests that there is a risk of false positive diagnosis that must be weighed against the risks and inconvenience of endoscopy. There is also a paucity of data in patients with Type 1 diabetes, where elevated tTG-IgA titres have sometimes been found to resolve spontaneously without GFD and/or a formal coeliac diagnosis [16]. The no-biopsy approach should therefore be even more thoughtfully applied in asymptomatic populations without factors increasing baseline risk or those with Type 1 diabetes.

## 5. Management and Follow-Up

A gluten-free diet (GFD) is the only treatment at present available for children with CD. Following diagnosis, children commence a lifelong, strict GFD, with families meeting with a paediatric dietitian for education on how to follow the diet. Following this, the frequency of dietetic follow-up is adjusted to suit the needs of the patient and their family. This is also guided by the presence of any nutritional deficiencies or risks, and by the resolution of clinical/serological parameters. Where possible, flexible open access to a paediatric dietitian will allow for monitoring and assistance with any problems or changes.

In an initial consultation, families will be counselled on naturally gluten-free foods as well as specifically manufactured gluten-free alternatives, and to think through the practicalities of a GFD that is suitable and nutritionally complete based on taste, as well as family and cultural habits. The risk of cross-contamination and the need to use separate cutlery, chopping boards, and other kitchen utensils to those used with gluten-containing food should be discussed.

Pure uncontaminated oats (those not contaminated with gluten and labelled ‘gluten-free’) are well tolerated by most patients with CD. However, they can still cause a reaction in approximately 5% of patients due to the presence of avenin protein, which is structurally similar to gluten [17,18]. Therefore, consideration to excluding all oats from the diet should generally be given until the symptoms and serology have normalised. At that stage, ‘gluten-free’ oats may be re-introduced while monitoring for any return of symptoms.

Lactose avoidance is recommended for clinical suspected (secondary) lactose intolerance, although this is mostly not required.

A full formal nutritional assessment including routine anthropometry, documentation of pubertal status, and biochemical evaluation of bone health (with vitamin D), along with full blood count, iron studies, vitamin B12 and folate assessment, is advisable with treatment as appropriate. Screening for associated autoimmune conditions, including type 1 diabetes, autoimmune thyroid and liver diseases, and Addison’s disease, should be considered, as these conditions may not be clinically apparent.

Patients with CD may have a degree of splenic dysfunction, putting them at higher risk of invasive pneumococcal disease [19,20]. Pneumococcal vaccination status should be determined at baseline, but there is a theoretical benefit to offering all patients with CD an additional pneumococcal polysaccharide vaccine (PPV), because it covers a greater breadth of serotypes than the conjugate vaccines administered in the routine schedule.

Children and young people with CD have increased rates of psychological symptoms compared to the general population, and difficulties adjusting to and maintaining long-term GFD are not uncommon [21,22]. Psychological input is considered a key part of the multidisciplinary management of CD in childhood. Increased knowledge about CD and membership of the CD society appear to be important factors in improving adherence to GFD and should be encouraged [23].

Screening of all first-degree relatives with serum tTG should be undertaken promptly after diagnosis, as households may reduce gluten intake to avoid the risk of cross-contamination for the affected individual.

Management and follow-up guidance on paediatric CD have been published by ESPGHAN [24]. The guidance suggests a follow-up at 3–6 months after diagnosis with follow-ups every 6–12 months thereafter. A serological follow-up should be carried out after 6 months, then at 12 months, and annually thereafter [25]. Paediatric patients who are lost to follow-up often have poor adherence to GFD and persistently positive serology [26]. One of the key markers of success and effective treatment in paediatric patients is growth [27].

Classical follow-up methods, such as serology testing, dietetic interviews, questionnaires, and endoscopy, have been shown to be inefficient, invasive, or inaccurate at evaluating adherence to a GFD [28]. The detection of gluten immunogenic peptide (GIP) in urine and stool is a novel test, and recent studies have examined whether it has a role in monitoring and follow-up in CD, specifically regarding its use as a tool for monitoring adherence to GFD. A recent systematic review evaluated 15 publications on the use of excreted GIP and found that GIP in urine/stool is a precise approach for measuring voluntary or involuntary gluten ingestion in patients with CD preventing future complications associated with gluten exposure [28]. It therefore has a potential role in monitoring adherence to GFD in paediatric patients.

## 6. Novel Therapies

A strict gluten-free diet remains the only treatment for CD. Though this is a safe and effective therapy, compliance with GFD can be challenging owing to many factors, including the taste and texture of non-gluten alternatives, social reasons, peer pressure, and the expense of obtaining GF foods [29]. Adherence to GFD remains a major hurdle, with studies reporting that approximately 25% of children with CD are non-adherent to GFD [30,31]. Left untreated, CD will lead to long-term consequences, including nutritional deficiencies, iron deficiency anaemia, poor growth, delayed puberty, mental health problems such as depression and anxiety, osteoporosis, osteopenia, malignancy [32] and increased risk of developing other autoimmune conditions, including Type 1 diabetes and autoimmune thyroid and autoimmune liver diseases [33].

Moreover, unintentional gluten exposure is also common, due to the ubiquitous nature of gluten, food cross-contamination, and hidden sources of gluten in non-food products such as medications and toothpaste. These factors, combined with our improved understanding of the pathogenesis of CD, has provided context for the development of treatment strategies beyond GFD. There is ongoing research in this field. The therapeutic options for CD currently being investigated can be broadly grouped into the following strategies:

1. The removal of or reduction in toxic gluten peptides before reaching the intestine, e.g., the genetic modification of gluten-containing cereals, microbial gluten modification like pre-treatment with microbial transglutaminase, masking of the antigenic capacity of gluten using polymeric resins, and luminal gluten detoxification using endopeptidases, gluten hydrolytic enzyme cocktails, and other agents.
2. Regulation of the immune-stimulatory effects of toxic gluten peptides, e.g., the inhibition of transglutaminase, blocking HLA DQ from binding to T cells, blocking lymphocyte recruitment, and the use of anti-cytokines.
3. The modulation of intestinal permeability: barrier-enhancing therapies like Larazotide acetate.
4. Immune modulation and the induction of gluten tolerance, e.g., Nexvax 2, hookworm infection.
5. The restoration of imbalance in the gut microbiota: probiotics/microbial therapies.

In the following section, we have attempted to summarise some of the important agents being investigated as non-dietary therapeutic agents for CD. The provision of a detailed description of the various agents currently under investigation is beyond the scope of this paper. Readers are referred to recent excellent reviews on this subject [34,35,36,37,38].

### 6.1. Endopeptidases

Latiglutenase (formerly ALV-003) acts to breakdown gluten before it passes into the small bowel. Phase II clinical trials have demonstrated a reduction in mucosal injury following a 6-week gluten challenge, with symptomatic improvements in bloating and tiredness, but not abdominal pains and constipation, when compared to the placebo group.

STAN-1 is another endopeptidase being explored as a possible therapeutic option. STAN-1 is a combination of enzymes thought to degrade gluten before it is absorbed into the gastrointestinal tract. Studies to date have been limited and, thus far, have not demonstrated any difference in STAN-1 compared with a placebo.

Similarly, Aspergillus Niger (AN-PEP), an additional endopeptidase, has been well tolerated in initial studies; however, no significant impact on disease behaviour was noted compared to the placebo group. This study was conducted over a short duration with a small sample size, so it remains to be seen whether this could still be a potential option.

Although more research is needed to further evaluate the therapeutic potential of endopeptidases, studies have shown promising evidence for both mucosal healing and symptom improvement, particularly with Latiglutenase, making it a potential contender for the future non-dietary treatment of CD [39,40,41].

### 6.2. Gluten-Sequestering Polymers (BL-7010)

The concept of sequestering gluten before it enters the small intestine is being explored. A phase I/II trial has been completed looking at the safety and systemic exposure of BL-7010. This is a synthetic polymer reported to have a high affinity for α-gliadin peptides. The proposed mechanism of action is sequestering gliadin and preventing its breakdown into immunogenic peptides. Pre-clinical studies have shown its potential effectiveness; however, further research is needed to evaluate this therapy given the possibility of the polymer binding to other medications [42].

### 6.3. Tight Junction Modulator—Larazotide Acetate (LA)

Larazotide acetate is shown to improve tight junction integrity, preventing gliadin-induced permeability, and therefore reducing inflammation of the small intestine. LA is a synthetic octapeptide, with a sequence analogous to a portion of the *Vibrio cholera* zonula occludens toxin. Multiple phase I and II trials have been carried out involving LA. Results have shown that the therapy has been well tolerated and has promising effects on symptom improvement (e.g., diarrhoea), but no effect in terms of histological improvement [43]. However, given its excellent safety profile and its efficacy, at a low dosage, in reducing symptoms in patients with non-responsive CD, larazotide acetate is expected to move forward to a phase III registration study [44].

### 6.4. HLA-DQ2 and HLA-DQ8 Blockers

The role of HLA in CD has been well established; the majority of patients with CD carry the Human Leucocytes antigens DQ2 or DQ8, which play a role in the activation of the immune response leading to inflammatory changes. HLA blockers have therefore been considered as an avenue of research for a potential new therapy for CD. Their evaluation remains in the pre-clinical phase; it is as yet unclear whether this may be a safe and efficacious treatment.

### 6.5. Tissue Transglutaminase 2 (TG2) Inhibitors

TG2 is a key part of the immune activation pathway leading to intestinal inflammation in CD. Therefore, TG2 inhibition may be a potential target for developing therapies, by preventing the presentation of gluten peptides by HLA-DQ2 and HLA-DQ8. To examine this concept, a study by Rauhavirta et al. looked at two TG2 inhibitors preventing the toxic effects of gliadin and obtained promising results [45].

More recently, a new drug, ZED1227, has been developed as a direct and specific inhibitor of TG2 in CD. Phase I trials have shown the drug to be safe and well tolerated. A further study has shown that three doses of ZED1227 protected coeliac patients from mucosal inflammatory damage. Phase 2b studies are pending, but this is a promising arm of future research and potential therapy [46].

### 6.6. Probiotics

The intestinal microbiota is known to play a significant role in health and disease. Dysbiosis has been seen to be associated with a variety of chronic inflammatory disorders, including in CD. Probiotics are not expected to provide a rapid cure for this complex disease, but rather, to alleviate the severity of symptoms [47]. To date, evidence suggests that manipulation of the gut microbiota has an important adjunctive role in the treatment of CD; further research is needed to evaluate probiotics as an independent effective therapy.

### 6.7. Gluten Tolerisation and Immunomodulation Therapies

Nexvax2 is a vaccination suggested to desensitise patients against gliadin peptides. It has passed phase 1 trials, despite having significant gastrointestinal side-effects of abdominal pain and vomiting. It has been suggested that the side-effects indicate immune activation similar to that in response to oral gluten ingestion. There is potential for the use of Nexvax2 as a future therapy for CD, but further research is needed [48].

### 6.8. Hookworm Infection

Intestinal parasitic infections are found to help regulate the immune system and prevent autoimmune and allergic diseases. The Hygiene Hypothesis suggests that restricting exposure to intestinal parasites in early childhood in the developed world has led to an increase in autoimmune diseases. A study by Croese et al. looked at introducing the hookworm parasite to patients with CD and assess its effects on intestinal damage and symptoms with a 52-week gluten challenge. There was no reduction in villous height-to-crypt depth, quality of life improved, and the tTG titre decreased despite escalating doses of gluten [49]. Although this is a potentially promising avenue of research, it is a treatment that is unlikely to be embraced by patients in the near future.

### 6.9. Nanoparticle

Nanoparticle is a trial product that proposes developing gluten tolerance via non-inflammatory mechanisms. In a Phase I study in mouse models with gliadin sensitivity, the Nanoparticle TIMP-GLIA induced unresponsiveness to gliadin and reduced markers of inflammation and enteropathy [34]. Clearly, this is research in its early stages, and more data are required.

### 6.10. Cathepsin S Inhibitor

Cathepsin is a lysosomal cysteine protease that plays a role in antigen presentation to MHC Class II. A drug named RO5459072 is being used in a Phase I trial [NCT02679014] to investigate whether it has a role in CD by measuring its response to a gluten challenge. The study aims to examine the pharmacokinetics, pharmacodynamics, tolerability, and safety profile of the drug; the results are pending.

### 6.11. Immune Cell-Targeted Therapies

Tofacitinib is an IL-15 antagonist (via pan-JAK inhibition) and is currently used in the treatment of inflammatory conditions. Trials are underway to investigate whether this has a role in the treatment of refractory CD. Likewise, the monoclonal antibodies AMG 714 and Hu-Mik-β-1 are also currently undergoing trials for potential use in refractory/non-responsive CD. IL-10 antagonists have also been considered in patient populations of refractory CD [35,36,37,38].

The CD-3 antigen is part of the T-cell receptor complex that helps to activate both T helper cells and cytotoxic T cells. Anti CD-3 antibodies should, in theory, help reduce inflammation in CD. In vitro studies have shown anti CD-3 agents may promote tolerance to gluten; however, published data are lacking [35,36,37,38].

## 7. Conclusions

CD is increasing in prevalence, partly due to increasing awareness and the sensitivity of diagnostic testing. Diagnostic algorithms are evolving, and the no-biopsy approach is being increasingly used, but this needs to be considered carefully, with a clear understanding of the guidelines and informed consent from parents/children. At present, a strict gluten-free diet is the only treatment option available. However, the search for alternative strategies for treatment is very active, as summarised in this review, but no studies have yet replaced the need for GFD. It is hoped that effective alternative treatments will emerge in the coming decades, which will be a welcome change for the CD population.

## Figures and Tables

**Table 1:** Gastrointestinal and Extra gastrointestinal manifestations of CD.

Persistent diarrhoea; Persistent constipation; Other persistent unexplained abdominal or gastrointestinal symptoms (e.g., pain, bloating, vomiting); Unexplained anaemia, iron deficiency unresponsive to treatment, or vitamin B12 or folate deficiency; Recurrent aphthous stomatitis; Faltering growth or idiopathic short stature; Delayed puberty, amenorrhoea; Dental enamel defects; Rickets, osteoporosis or pathological fractures; Arthritis or arthralgia; Unexplained raised transaminases or liver disease; Prolonged fatigue, weakness, or irritability; Unexplained neurological symptoms (e.g., palsies, neuropathies, migraine, ataxia); Dermatitis herpetiformis; Epilepsy with cerebral calcification.

**Table 2:** Conditions with increased risk of CD.

Type I diabetes (~5% prevalence of CD); Autoimmune thyroid disease (~6% prevalence in children, who may be hyper- or hypothyroid); Down Syndrome, Turner Syndrome (both 5–10% prevalence), Williams Syndrome (8.2%); Selective IgA deficiency (magnitude of association uncertain); Autoimmune liver disease; Addison’s disease and autoimmune polyglandular syndromes; First-degree relatives of patient with CD (prevalence in the region of 10%).

## Data Availability

No new data were created or analysed in this study.
